# Supplementary material for: Developing a novel force forecasting technique for early prediction of critical events in robotics
Source: PLoS One. 2020 May 7;15(5):e0230009. doi: 10.1371/journal.pone.0230009 (PMC7205263; doi:10.1371/journal.pone.0230009)
Supplement: Attachment — Submitted filename: Response to Reviewers.pdf [file pone.0230009.s001.pdf]

Response to Reviews

We tried to address all the comments and suggestions by the academic editor and the reviewers to the best of our ability. Any changes made in the revised manuscript addressing the comments have been marked in blue color. Below are our responses to the reviewer comments marked in italic. Please note that the section numbers mentioned in our response correspond to the revised manuscript.

Reviewer #1: This paper proposes a novel CFDL-MFP method to predict time series data, and develops a needle bucking prediction algorithm based on the predicted data. With the proposed method, the accuracy and the real-time performance of the critical event prediction are greatly improved. Corresponding simulations and experiments are implemented to evaluate the proposed method. This paper is worth publishing in PLOS ONE after minor revision.

A few suggestions to improve the quality of the paper:
1. The Organization of the paper should be adjusted. The method should be described first, and the experimental evaluation should be given following the proposed method. So the organization of the paper should be sections 3, 5, 4, 6,7 in turn, and what is more, section 6,7 should be merged into one section.
Response: *We thank the reviewer for this suggestion but would like to explain why 3, 4, and 5 are ordered the way they are. Section 3 describes the theory behind the development of the novel forecasting algorithm. Section 4 describes performance results of the forecast algorithm compared to existing forecast technique (ARIMA), which is precursor to the development of an event prediction algorithm in Section 5. Since the forecast results motivate the development of buckling prediction algorithms as explained in Section 4.3.2, we decided to maintain the original order for flow of the content. For clarity, we now added the point "Section 4 discusses simulation results of the proposed forecast as precursor to buckling prediction." in Section 1.3. We agree that sections 6 and 7 can be merged and have done so. We thank the reviewer for this suggestion.*

2. It would be better if the proposed CFDL-MFP needle prediction algorithm could be evaluated on real clinical data. If so, the paper will be more convincible and have a bigger impact.
Response: *We thank the reviewer for this suggestion. Clinical data would be ideal but very challenging to obtain using our research robot due to lengthy required IRB or IACUC approvals to do human or animal studies. Because the main focus of the paper was to introduce a novel forecasting technique with low computation times and high accuracy, we chose to evaluate using simulation, artificial tissues (which are commonly used in needle steering studies), as well as ex vivo biological tissue (which is important but less commonly used). In section 4, we do show that ability to predict needle insertion force from data we found in the literature of needle insertions into ex vivo human liver. We note that PI Majewicz Fey has extensive experience with needle steering studies in both ex vivo and live animal tissue from prior work – to our knowledge there is no data on needle steering in live humans. In the future, we agree that proving our algorithms using clinical data, even if it's not needle steering but more standard needle insertion tasks, would have high impact and is something that we are evaluating in terms of feasibility.*

3. What is the extensibility of the proposed method? Could the proposed prediction method also be applied for other event prediction problems?

Response: Yes, we believe this is a strength of our proposed method. First, the force forecasting method can be applied to any time-series data. For example, section 4 shows how we can predict force data from straight needle insertion in human liver, but if some other event required measuring position data or other types of data, our algorithm could have been used equally well. To predict events, all that is required is some kind of model or pattern to determine when a particular event occurs. For our current work, needle buckling can be characterized by force increases. In our prior work, we have also predicted events such as needle curvature changes and tissue displacement using our prior knowledge of the patterns found in both force and position data during these events (see citation below). We are currently working on integrating our prior event prediction methods with our new data forecasting technique.

Narayan, M., Fey, A.M. and Choti, M.A., 2018. Data-driven detection of adverse events in robotic needle steering. In *2018 International Symposium on Medical Robotics (ISMR)* (pp. 1-6). IEEE.

Reviewer #2: In this paper, the authors presented an interesting and novel prediction method with good accuracy and low computational time, which make the method applicable in real time computations. The forecast algorithm is used to predict needle buckling by the axial force and the needle-tip position data.

The paper is organized very well, the literature review is very complete, and the prediction model is presented with details. The results show that the proposed method can predict the needle buckling even in reality.

Suggestion: The authors concluded that the prediction results did not depend on the tissue type. It seems that in the buckling prediction logic, the tissue stiffness has not been considered. I suggest the authors to mention it as an assumption (if necessary).

Response: Yes, it is true in some sense that our algorithm does not explicitly include tissue stiffness in the detection and this is completely by design. Tissue stiffness properties are highly variable between patients, types of organs, and can even change during a surgical procedure (e.g., tissue ablation will make tissue stiffer). Hence, we have put considerable effort into designing our detection algorithms in a way that is generalizable enough to work across all these different tissue parameters. This is accomplished through the use of a generalizable sigmoid detection metric (which can be found in detail in our prior work, citations below), which instead identifies only significant changes in the real-time force data.

Narayan, M., Choti, M. A. & Fey, A. M., 2019. Data-driven detection of needle buckling events in robotic needle steering. *Journal of Medical Robotics Research,* 04(02).

The reviewers comment however did help us realize an important limitation of our work which we did not explicitly state and that is that would algorithm can only detect that an event occurs and not the cause of the event (e.g., distinguishing buckling due to a stiffer portion of tissue or due to an rigid obstacle). For more clarity, we now added the first sentence in the last paragraph of Section 7 of revised manuscript: "One limitation of our proposed algorithm is that we can only distinguish when buckling events occur, but not necessarily the cause of those events (e.g., a stiffer portion of tissue vs. a rigid obstacle)."

Question: It seems that the tip-position and the axial force have been used for buckling prediction. It is not clear for me how you used the force and position data together for prediction. Could you explain it?

Response: Essentially, the algorithm works by detecting when a high force increase event occurs, while also having a reduction in needle tip position. The details are a little more complex because the data is normalized in a way to enable generalization of prediction across various tissue types but can be found in our prior work. The discussion of how we use axial force and needle-tip position data can be found in Section 5.1 Prediction Methods. We also clarified in Section 6.2 that the plots shown include both force and position data.
